# Digital Homework Support Program for Children and Adolescents With Attention-Deficit/Hyperactivity Disorder: Protocol for a Randomized Controlled Trial

**DOI:** 10.2196/44553

**Published:** 2024-11-21

**Authors:** Fanny Gollier-Briant, Laurence Ollivier, Pierre-Hugues Joalland, Stéphane Mouchabac, Philippe Leray, Olivier Bonnot

**Affiliations:** 1 University of Nantes Nantes France; 2 Centre Hopsitalo-Universitaire of Nantes Nantes France; 3 Capacités SA Nantes France; 4 University of Nantes - Laboratoire des Sciences du Numérique Nantes France; 5 Sorbonne Université-infrastructure for Clinical Research In Neurosciences Psychiatrie Paris France; 6 Assistance Publique - Hôpitaux de Paris St Antoine Hospital Paris France; 7 Paris-Saclay University Kremlin Bicêtre France; 8 Barthélémy Durand Hospital Sainte Geneviere des Bois France

**Keywords:** attention-deficit/hyperactivity disorder, mobile app, assisted homework session, digital mental health, e-health, smartphone, psychiatry, neurodeveloppemental disorders

## Abstract

**Background:**

Attention-deficit/hyperactivity disorder (ADHD) affects 4% to 5% of the general population. Homework sessions are frequent conflictual moments characterized by increased anxiety in children and stress in their parents, contributing to a lower family quality of life. Children with ADHD experience more severe homework problems than typically developing peers. Poor academic performance in individuals with ADHD is partly attributed to challenging homework. Psychoeducational and school-based approaches are time-consuming and not fully accessible to professionals. Digital tools, such as smartphone and tablet apps, might offer an interesting alternative. We present our digital homework support program for children and adolescents, known as “Programme d’Aide Numérique aux Devoirs pour Enfant avec TDA-H” (PANDAH), along with the study protocol of our ongoing randomized controlled trial.

**Objective:**

This study aims to test PANDAH’s efficacy in improving homework performance and family quality of life.

**Methods:**

Individuals aged 9-16 years with an ADHD diagnosis and no comorbid psychiatric disorders are included. This is a multicenter study involving 9 reference centers for ADHD in France. The study comprises (1) a 3-month period with a randomized controlled trial design, where participants are divided into 2 parallel groups (group 1: care as usual or waiting list; group 2: PANDAH app), followed by (2) an extension period of 3 months (months 3-6), during which all participants will have access to the app. This second phase serves as a crucial incentive for patients initially randomly assigned to group 1. Assessments will be conducted at baseline, month 3, and month 6 for each patient by trained psychologists. The primary end point will be the global Homework Performance Questionnaire (HPQ), Parent version score at 6 months. The main analysis will adhere to the “intent-to-treat principle” (all patient data will be analyzed according to their initial group determined by randomization). We expect (1) HPQ score improvement in individuals using the app during the first 3-month period compared to individuals not using the app; (2) greater HPQ score improvement for individuals using the app for 6 months compared to those using the app for 3 months only; and (3) adherence to the PANDAH program, measured with in-app metrics.

**Results:**

Recruitment began in January 2024, and the trial is ongoing.

**Conclusions:**

This study contributes to the digital transformation of health care. The use of smartphone apps in self-care and self-management is a societal phenomenon, and its implementation in the field of psychiatry is of particular interest. The app might serve as both valuable support for patients and an opportunity for parents to distance themselves from conflict-laden homework sessions. Since the market for smartphone apps in the health care and well-being sector is primarily industry driven, it is crucial to have an academic conception and evaluation of such digital tools.

**Trial Registration:**

ClinicalTrials.gov NCT04857788; https://clinicaltrials.gov/ct2/show/NCT04857788

**International Registered Report Identifier (IRRID):**

PRR1-10.2196/44553

## Introduction

Attention-deficit/hyperactivity disorder (ADHD) affects 4% to 5% of the general population [1,2]. Elementary school children with ADHD face severe academic challenges characterized by reduced seatwork completion, seatwork accuracy, on-task behavior, and homework performance compared to their peers [3,4]. Homework sessions are often tense moments marked by increased anxiety in children and stress in their parents, undeniably contributing to decreased family quality of life. Children with ADHD experience more severe homework difficulties than typically developing peers [5,6]. Longitudinal studies also indicate that homework performance during elementary school serves as a key predictor of later academic success for individuals with ADHD [7].

These issues persist into later school years, with middle and high school students with ADHD exhibiting poorer organizational skills, lower grades, increased truancy, and higher rates of suspension and grade retention compared to their peers [8-11]. Moreover, significantly fewer individuals with ADHD graduate from high school (68% vs 100% of controls) [11] or enroll in 4-year colleges and universities compared to controls (29.5% vs 76.8% of controls) [12]. In summary, academic underachievement is strongly associated with ADHD [13-19], and homework sessions could be a critical area of intervention to prevent this outcome.

To address this issue, several health care interventions are available, such as classroom management and behavioral consultations in school settings [20-22]. Although classroom management procedures and interventions have demonstrated short-term improvements in classroom behavior and seatwork completion and accuracy among children with ADHD in classroom and analog classroom settings [23-25], it is potentially beneficial to explore additional interventions targeting the academic functioning of children with ADHD, both at home and the parenting level. Research indicates a positive relationship between homework and achievement, with a stronger link observed in middle and high school [26].

Among the various behavioral parent training (BPT) programs for children with ADHD, two programs have focused on homework functioning: (1) the Family School Success Program [27] and (2) Parents and Teachers Helping Kids Organize [28]. Both programs incorporate effective components of BPT, such as consistent responses, time-out, goal setting, rewards, and positive attention. They have demonstrated efficacy in improving parent-reported problematic behaviors during homework time, with moderate to large effect sizes ranging from 0.52 to 1.51 [28,29]. Furthermore, these academically focused BPTs have improved self-reported parenting strategies, teacher-reported homework problems, and parent- and teacher-reported organizational skills. Interestingly, children on stimulant medication who received BPT showed greater improvement in parent-reported homework management than their unmedicated peers [29]. However, none of these programs have incorporated new technologies thus far.

Over the past 4 years, we have been developing a smartphone app to assist in homework management within therapeutic groups, aiming to help both parents and patients improve their homework sessions and concentration. However, the real and perceived use of an app significantly impacts its usefulness [30]. Therefore, to be efficient and reduce attrition, a digital tool needs a specific design; an intuitive user interface; and the addition of an artificial intelligence module to create an algorithm that can analyze, predict, and provide suitable personalized advice. As a result, we developed *Programme d’Aide Numérique aux Devoirs pour Enfant avec TDA-H* (PANDAH; “a digital program to help children with ADHD during homework sessions” in French) to support children with ADHD during their homework sessions with minimal or no parental assistance.

This study aims to investigate the impact of PANDAH on children and adolescents aged 9 to 16 years with ADHD over a 6-month period, focusing on homework performance and well-being.

## Methods

### Recruitment

This multicenter study involves 9 reference centers for ADHD in France, which are specialized centers covering specific geographic areas with multidisciplinary teams experienced in ADHD diagnosis and treatment. We plan to include a total of 360 patients with ADHD from waiting lists of reference centers (median waiting time in France is 9 months) [31]. The inclusion and exclusion criteria are shown in Textbox 1.

**Inclusion and exclusion criteria.**

**Inclusion criteria**

- Patients aged 9-16 years, currently on the waiting list of one of the participating centers
- Consensus diagnosis of attention-deficit/hyperactivity disorder (ADHD) by 2 mental health care professionals
- Written informed consent provided by patients and their parents

**Exclusion criteria**

- Patients with severe psychiatric conditions such as schizophrenia, bipolar disorder, or autism spectrum disorders, along with suicidal ideation or behavior (assessed by the Child Behavior Checklist Scale)
- Patients with no access to a smartphone or limited smartphone access (eg, patients in social facilities or those with low economic status)
- Patients with mild to severe mental retardation or deemed clinically unable to use the app
- Patients participating in special programs beyond standard care received while on the waiting list
- Patients not attending school (no homework)
- Patients in the titration period of methylphenidate or those with unstable doses over the past 3 months

### Study Design

**Figure 1:**
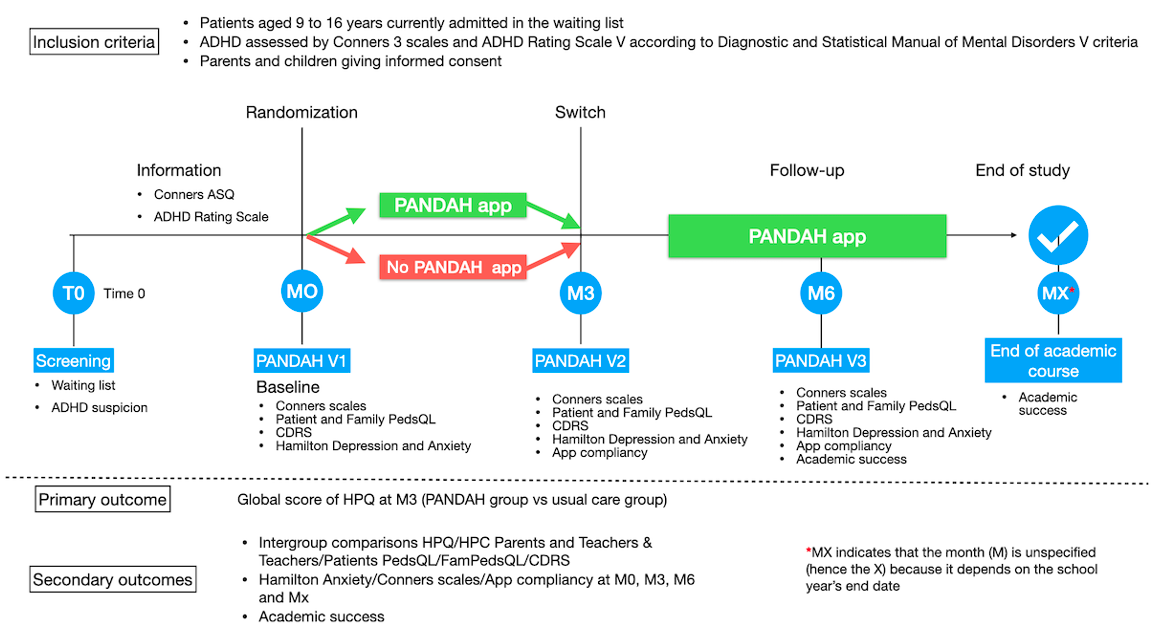
Study design of PANDAH protocol. ADHD: attention-deficit/hyperactivity disorder; ASQ: Abbreviated Symptom Questionnaire; CDRS: Children’s Depression Rating Scale–Revised in Adolescents; HPC: Homework Problem Checklist; HPQ: Homework Performance Questionnaire; M: month; PANDAH: Programme d’Aide Numérique aux Devoirs pour Enfant avec TDA-H; PedsQL: Pediatric Quality of Life Inventory.

The primary investigator’s center is the Department of Child and Adolescent Psychiatry at Nantes University Hospital Centre, which includes a clinical research unit in psychiatry, addictology, and child and adolescent psychiatry. The study will consist of a 3-month period with a randomized controlled trial design, where participants are divided into 2 parallel groups (group 1: care as usual or waiting list; group 2: PANDAH app). This is followed by a 3-month extension period (month 3 [M3] to month 6 [M6]), during which all participants will have access to the app (Figure 1). This second phase serves as an incentive for patients initially randomized into group 1. Assessments will be conducted at baseline (M0), M3, and M6 for each patient. Baseline evaluations will be performed by trained psychologists at inclusion and will include age, sex, school grade, treatment history, Child Behavior Checklist, Hamilton Anxiety Scale, ADHD Rating Scale, and Homework Performance Questionnaire (HPQ) [32] with parents. Interviews will last approximately 2 hours and 30 minutes, including instructions for using the app for group 2. Evaluations at M3 and M6 will be similar (2 hours and 30 minutes), allowing the assessment of the maintenance of effects and efficacy.

A screening visit will be scheduled for patients provisionally diagnosed with ADHD based on the psychologist’s interpretation of *Conners 3rd Edition* parents’ and teachers’ scales and ADHD Rating Scale V. The screening visit will involve evaluation by 2 health care professionals who will reach a consensus on the diagnosis. If the ADHD diagnosis is confirmed and the inclusion and exclusion criteria are met, participants will be fully informed about the study, their questions will be addressed, and consent (from both parents and children) will be obtained. For group 2 participants, the investigator will assist with setting up the app (15-20 minutes). Technical support will be provided remotely by each center from 8:00 AM to 6:00 PM via phone or email.

Given the unique attributes of our app, a traditional randomized controlled trial design proved unfeasible. Instead, we adopted a crossover design to facilitate a comparison between our app and a null intervention scenario. While this approach is likely to yield positive results, it may not definitively establish the specific efficacy of our intervention. Subsequent analysis will encompass the entire cohort, allowing for a comparison between those who used the app for 3 months and those who used it for 6 months. Any noticeable disparities may serve as an indicator of our intervention’s efficacy.

### Intervention

**Figure 2:**
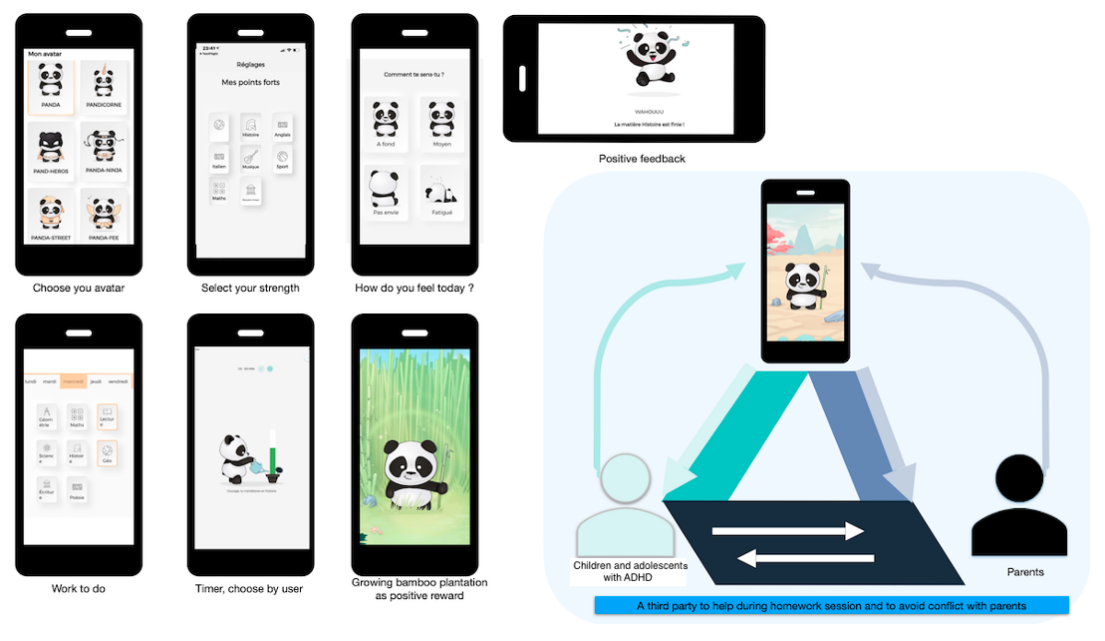
Screenshots and general concept of the PANDAH app. ADHD: attention-deficit/hyperactivity disorder; PANDAH: Programme d’Aide Numérique aux Devoirs pour Enfant avec TDA-H.

The intervention is based on the PANDAH app, which engages patients in understanding their grades, homework location, surrounding environment, habits, schedules (including sports and therapy), and expectations. PANDAH guides students throughout their homework sessions, from ensuring they have everything they need to create a comfortable workspace to packing for the next day. During sessions, patients receive positive feedback and reinforcement when appropriate. The app also provides personalized artificial intelligence–enhanced advice, such as suggesting students tackle challenging subjects first to reduce anxiety and avoid extended sessions. PANDAH is used independently at home but is complemented by real-life sessions (3 times a year during the school term) to familiarize children and adolescents with the app and address any questions (Figure 2).

Usage during the study will be monitored in terms of time, frequency, and schedule, with in-app data securely transferred to the principal investigator for analysis.

### Primary End Point

The primary end point will be the global score on the HPQ, Parent version (HPQ-P) [32]. This 12-item questionnaire, developed in partnership with parents using focus groups and interviews, uses a 4-point scale.

### Secondary End Points

The secondary end points include (1) global and domain scores of HPQ-P at M6 [32]; (2) global and domain scores of HPQ, Teacher version at M3 and M6 [32]; (3) Patient Pediatric Quality of Life Inventory and Pediatric Quality of Life Inventory Family Impact Module at M3 and M6 [33,34]; (4) global score of Homework Problem Checklist for Parents at M3 and M6 [35,36]; (5) Inattention and Hyperactivity scores from Conners 3 at M3 and M6 ; (6) in-app scores for fill rate and number of complete app sessions during the M0-M3-M6 period; (7) Children’s Depression Rating Scale–Revised in Adolescents and Hamilton Anxiety Scale at M3 and M6; and (8) Likert scale for teachers at M3, M6, and end of the academic year (Mx) regarding changes in students’ academic levels: “Degraded/Slightly Degraded/Slightly Improved/Improved” [37].

Secondary criterion 6 will further allow us to assess the actual usage of the app by estimating the time the app is open and the time of interaction (using fingers) with the app. This way, we can evaluate whether the child is not solely focused on the app but engaged in other activities as well.

### Safety

Data security is a crucial aspect of digital tools. Most data will be stored on the user’s smartphone and inaccessible to unauthorized individuals without the user’s personal code. Notably, the app does not require an internet connection to function. Patients included in the research will transfer data securely to investigators using a multistep process, ensuring encryption and secure handling. The data will be decrypted and analyzed only by the principal investigator. Data will be securely stored and analyzed in a Microsoft Excel spreadsheet.

### Statistical Analysis

The primary analysis will adhere to the “intent to treat principle,” analyzing all patient data based on their initial group determined by randomization.

Descriptive statistical analyses will be conducted for categorical data (number and percentage) and continuous data (range, mean, median, and IQR).

Confirmatory factor analysis will be applied preliminarily to the HPQ-P and HPQ, Teacher version questionnaires to assess construct validity. Adequate fit will be considered if the Comparative Fit Index≥0.90 and the root mean square error of approximation≤0.08. Meaningful differences between well-fitting models will be assessed using Δ Comparative Fit Index>+0.01. Reliability will also be assessed using Cronbach α, considered satisfactory if above 0.7.

Cross-sectional comparisons (eg, M3) of continuous and categorical data will use linear or logistic regression, adjusting for M0 if appropriate based on the data collection schedule.

Longitudinal data will be analyzed and compared between the 2 groups using linear mixed models with random effects to account for repeated measurements within patients. Different models, including random intercept or slope models, will be assessed based on the Akaike information criterion, and normality and homoscedasticity of residuals will be examined. A dummy variable reflecting the switch at M3 from the control group to the PANDAH app group will be included in the models (M0 to M6).

Assuming a mean difference of 0.4 on the global score of the HPQ-P at M3 between the 2 groups, with a common SD of 0.9, a total of 264 patients (n=132, 50% in each group) is required to detect this difference with a 5% type I error and 95% power in a 2-tailed *t* test. Assuming 0 attrition (no loss to follow-up) due to proximal assessments at M3, saturation of the child psychiatrist network, and no removal or relocation of families shortly after the school year begins, our aim is to include precisely 264 patients in the PANDAH trial.

### Ethical Considerations

Ethical approval was obtained on June 15, 2021, from the Comité de Protection des Personnes—Est V of CHU de Strasbourg, France, with reference: 21/19/SI 21.02.01.49939. Written informed consent was obtained from the patients and their parents. All personal data from the PANDAH app are fully encrypted and anonymous. There was no compensation for participants

### Expected Findings

We expect (1) improvements in HPQ score in individuals with ADHD using the app during the first 3-month period compared to individuals not using the app; (2) greater improvements in HPQ score for individuals using the app for 6 months compared to those using the app for 3 months only; and (3) adherence to the PANDAH program, measured with in-app metrics.

## Results

Recruitment began in January 2024, and the trial is ongoing.

## Discussion

### Overview

This study aligns with the digital transformation of health care, leveraging smartphone apps for self-care and self-management, which is particularly relevant for children and adolescents who are digital natives. Collecting data through smartphones, known as ecological momentary assessment, and using this data for ecological momentary intervention with algorithm-driven personalized patient feedback and advice is a burgeoning trend [38-42]. While there are existing apps for ADHD, they tend to lack intuitiveness and interactivity and focus mainly on treatment management, comorbid symptoms, or psychotherapy. Currently, there is only 1 Food and Drug Administration–approved therapeutic app for ADHD, primarily based on cognitive therapy.

The PANDAH app focuses on homework sessions and helps with planning and organization, providing a potentially more engaging and effective approach compared to adult supervision. It addresses the limitations of medication, which may have side effects and limited duration, especially during after-school hours. The most widely used evidence-based treatment for children with ADHD [43], psychostimulants, may not yield long-term academic gains [44,45]. Therefore, there is a need for psychosocial and psychoeducational methods to enhance educational outcomes for these children. Digital tools offer a promising alternative and may reduce the need for medication.

In summary, our aim is to evaluate the efficacy of a digital-only program, which could be a cost-effective complement to traditional ADHD care, especially considering the societal trend toward increasing use of health care apps. However, it is important to address technology access issues for low-income families who may not have smartphones.

### Conclusions

This study envisions that smartphone-based digital tools could be highly effective for the youth population due to several synergistic reasons: (1) daily care support that is more engaging than adult supervision; (2) personalized advice with positive reinforcement; (3) addressing organizational and time management skills; and (4) the strong connection between youth and smartphones. If proven effective, this digital-only program could enhance the academic achievement of patients with ADHD and pave the way for the digital transformation of ADHD health care protocols. However, it is crucial to address economic challenges related to technology access for low-income families in future health care plans.

## Appendix

Multimedia Appendix 1 Ethics committee approval letter.

## Abbreviations

ADHD: attention-deficit/hyperactivity disorder
BPT: behavioral parent training
HPQ: Homework Performance Questionnaire
HPQ-P: Homework Performance Questionnaire, Parent version
M0: baseline
M3: month 3
M6: month 6
Mx: end of the academic year
PANDAH: Programme d’Aide Numérique aux Devoirs pour Enfant avec TDA-H

## Data Availability

Data sharing is not applicable to this paper as no datasets were generated or analyzed during this study. All data will be provided as supplementary material at the end of the study.
