# Supplementary material for: Digital Homework Support Program for Children and Adolescents With Attention-Deficit/Hyperactivity Disorder: Protocol for a Randomized Controlled Trial
Source: JMIR Res Protoc. 2024 Nov 21;13:e44553. doi: 10.2196/44553 (PMC11621713; doi:10.2196/44553)
Supplement: Multimedia Appendix 1 [file resprot_v13i1e44553_app1.pdf]

COMITÉ DE PROTECTION DES PERSONNES
« EST IV »

1, place de l'Hôpital
67091 STRASBOURG Cedex
☎: 03.88.11.60.03
Fax : 03.88.11.63.48
E-mail : cpp.est4@chru-strasbourg.fr

Strasbourg, le 15 juin 2021

Le Président

Madame A. OMNES
Responsable Département Promotion
CHU de Nantes
5 allée de l'Ile-Gloriette
44093 Nantes cedex 1

**Réf CPP / SI**	CPP 21/19 / SI 21.02.01.49939	Catégorie : 2 hors L5311-1
**Titre de la recherche**	Randomized controlled trial for efficacy assessment of a Program of Digital Accompaniment to optimize homework performances and increase Familial Quality of Life in 9 to 16 years children with ADHD / Évaluation de l'efficacité de l'application Smartphone PANDAH pour optimiser les performances des devoirs à la maison et augmenter la qualité de vie familiale chez les enfants de 9 à 16 ans atteints de TDAH. Essai contrôlé randomisé<br>Etude PANDA-H	
**Identification**	IDRCB 2021-A00261-40	
**Promoteur**	CHU de Nantes<br>5 allée de l'Ile-Gloriette<br>44093 Nantes cedex 1	
**Investigateur Coordonnateur**	Dr. Olivier BONNOT<br>Hôtel Dieu<br>30 boulevard Jean Monnet<br>Nantes	

Madame,

Je vous remercie pour votre réponse du 8 avril 2021, faisant suite à mon courrier du 25 mars 2021 concernant le projet de recherche référencé ci-dessus.

Les précisions apportées par le promoteur répondent aux attentes du Comité et j'ai donc le plaisir de vous adresser l'**Avis Favorable** du CPP Est IV pour votre demande examinée lors de sa séance du 16 mars 2021.

Je vous prie de croire, Madame, en l'assurance de mes salutations les meilleures.

Professeur Erik-André SAULEAU

PJ : 1

COMITÉ DE PROTECTION DES PERSONNES
« EST IV »

1, place de l'Hôpital
67091 STRASBOURG Cedex
☎: 03.88.11.60.03
Fax : 03.88.11.63.48
E-mail : cpp.est4@chru-strasbourg.fr

Le Président

Séance CPP du 16 mars 2021

Avis émis le 15 juin 2021

A V I S

**Réf CPP / SI**	21/19 / SI 21.02.01.49939	Catégorie : 2 hors L5311-1
**Titre de la recherche**	Randomized controlled trial for efficacy assessment of a Program of Digital Accompaniment to optimize homework performances and increase Familial Quality of Life in 9 to 16 years children with ADHD / Évaluation de l'efficacité de l'application Smartphone PANDAH pour optimiser les performances des devoirs à la maison et augmenter la qualité de vie familiale chez les enfants de 9 à 16 ans atteints de TDAH. Essai contrôlé randomisé<br>Etude PANDA-H	
**Identification**	IDRCB 2021-A00261-40	
**Promoteur**	CHU de Nantes<br>5 allée de l'Ile-Gloriette<br>44093 Nantes cedex 1	
**Investigateur Coordonnateur**	Dr. Olivier BONNOT<br>Hôtel Dieu<br>30 boulevard Jean Monnet<br>Nantes	

Le Comité a été saisi le 1er février 2021 d'une demande d'avis pour le projet de recherche référencé ci-dessus, par

Madame A. OMNES
Responsable Département Promotion
CHU de Nantes
5 allée de l'Ile-Gloriette
44093 Nantes cedex 1

Composition du Comité lors de la séance

Etaient présents en séance du 16 mars 2021
mais n'ont pris part au vote que les membres titulaires et les membres suppléants en situation de représentant

	Catégories	Titulaires	Suppléants
**1er collège**	**P**ersonnes qualifiées en matière de recherche biomédicale, en biostatistique ou en épidémiologie	Prof. Philippe Hénon<br>Dr Philippe Lutun<br>Prof. Laurent Monassier<br>Prof. Erik-André Sauleau	Dr Dominique Astruc<br>Dr François Lefèbvre
	**M**édecins généralistes	Dr Guy Haberer	Dr Fabien Rougerie
	**P**harmaciens	Dr Anne-Cécile Gérout	/
	**I**nfirmières	/	Mme Anne-Marie Tosato
**2e collège**	**P**ersonnes qualifiées en matière d'éthique	M. Jean Degert	M. Vlad Titerlea
	**P**ersonnes qualifiées dans le domaine social	Mme Fabienne Barth Foltz	Mme Brigitte Marchal Bucquet
	**P**ersonnes autorisées à faire usage du titre de psychologue	Mme Nadine Fialon	/
	**P**ersonnes qualifiées en matière juridique	Mme Christine Gugelmann	Mme Catherine Burger
	**R**eprésentants des usagers	Mme Laurence Grandjean<br>M. Francis Louis Bouché	Mme Annie Nock

…/…

Après avoir analysé le protocole, le rapport BENEFICE/RISQUE du projet, les conditions d'information et de protection des participants, la présence des documents administratifs obligatoires et nécessaires à l'expertise, le Comité donne un

AVIS FAVORABLE

Liste des documents présentés sur lesquels l'avis du Comité est rendu :

Courrier de saisine du 26 janvier 2021 (déposé sur le SI le 01/02/2021)
Formulaire de demande d'AEC (FAEC) du 29/01/2021
Document additionnel à la demande d'avis du 29/01/2021
Document d'information du patient 9-14 ans, version 1 du 25/01/2020
Document d'information du patient 15-16 ans, version 1 du 25/01/2020
Document d'information des parents, version 1 du 13/01/2020
Formulaire de consentement du titulaire de l'autorité parentale d'un mineur ou du représentant légale d'un mineur sous tutelle, version 1 du 25/01/2021
Protocole en anglais, Version 1 du 25/01/2021
Résumé Version n°01 du 25/01/2021
Attestation d'assurance du 29/01/2021 (300 sujets, du 01/09/2021 au 30/08/2024)
Justification de l'adéquation des moyens du 26/01/2021
Liste des investigateurs Version 1 du 25/01/2021, 9 CV et 5 BPC
Récépissé CNIL MR-001 du 21/10/2016

Courrier de réponse du 07/04/2021
Document d'information du patient 9-14 ans, version 1 du 25/01/2020 (avec modifications apparentes)
Document d'information du patient 15-16 ans, version 1 du 25/01/2020 (avec modifications apparentes)
Document d'information des parents, version 1 du 13.01.2020 (avec modifications apparentes)
Formulaire de consentement du titulaire de l'autorité parentale d'un mineur ou du représentant légale d'un mineur sous tutelle, version 1.1 du 06/04/2021 (avec modification apparente)
Consentement, version 1.1 du 06/04/2021
Protocole, version 1 du 25/01/2021 (avec modifications apparentes)
Résumé, version 1.1 du 06/04/2021 (avec modifications apparentes)

Professeur Erik-André SAULEAU
Président du CPP Est IV
